# Functional Characterization of RseC in the SoxR Reducing System and Its Role in Oxidative Stress Response in *Escherichia coli*

**DOI:** 10.4014/jmb.2410.10007

**Published:** 2024-11-25

**Authors:** Kang-Lok Lee, Joon-Hee Lee, Yun-Hee Kim, Jung-Hye Roe

**Affiliations:** 1 Department of Biology Education, IALS, Gyeongsang National University, Gyeongsang National University, Jinju 52828, Republic of Korea; 2 College of Pharmacy, Pusan National University, Pusan 46241, Republic of Korea; 3 Laboratory of Molecular Microbiology, School of Biological Sciences, and Institute of Microbiology, Seoul National University, Seoul 08826, Republic of Korea

**Keywords:** Oxygen-sensitive Fe-S, RseC, SoxR reducer, Rnf, membrane protein

## Abstract

The reducing system of SoxR consists of a putative electron transfer system encoded by the *rsxABCDGE* operon, RseC encoded from the unlinked *rpoE-rseABC* operon, and ApbE. RseC is composed of two transmembrane helices, with both the N-terminal and C-terminal domains located in the cytoplasm. The N-terminal domain has a four-cysteine motif, CX_5_CX_2_CX_5_C, in the cytoplasm, with the latter three cysteines highly conserved in RseC homologs, allowing the SoxR reducer complex to function in *Escherichia coli*. These three cysteines can form an oxygen-sensitive Fe-S cluster when only the N-terminal domain is expressed in a truncated form. Without the C-terminal domain, RseC shows no significant difference in interaction with the SoxR reducer complex, but its ability to complement the function of an *rseC* mutant is greatly reduced. Additionally, the *rseC* mutant exhibits weak resistance to cumene hydrogen peroxide in the stationary phase and increased sensitivity to hydrogen peroxide in the exponential phase, independent of the SoxR regulon. This suggests that the full-length sequence of RseC is essential for its function and that it may have SoxR-independent additional roles.

## Introduction

Bacteria have various forms of electron transfer systems in cytoplasmic membrane. The Na^+^-translocating NADH:ubiquinone oxidoreductase (Nqr) and Complex I of the respiratory chain are well-studied energy conserving complex. Nqr and Complex I couple electron flow from NADH to quinone with the translocation of monovalent cation Na^+^ and proton outside cytoplasmic membrane, respectively. Rnf has some subunits that are homologous to Nqr, and is classified as an energy-conserving complex evolutionarily related to Nqr. [1]. Rnf, functioning as a membrane-bound ferredoxin:NAD^+^ oxidoreductase, serves multiple roles in anaerobic bacteria, including acting as a multifunctional respiratory enzyme involved in nitrogen fixation, carbon dioxide fixation, metabolism of low-energy substrates such as ethanol and lactate, and facilitating gut colonization in mice [2-8].

*Escherichia coli* has both Complex I of the respiratory chain and homologs of Rnf. Rnf homologs of *E. coli* reduce the [2Fe-2S] cluster of SoxR, a transcription regulator [9]. Redox active chemicals generate oxidative stress, which is sequentially regulated through the oxidation of the [2Fe-2S] cluster of SoxR and the activation of SoxS [10-13]. When stress is relieved by the activated *soxS* regulon, the transcription of *soxS* is restored to its original level by a SoxR reducer system [10, 11, 14, 15]. A reducing system of SoxR consists of an electron transfer system encoded by the *rsxABCDGE* operon, RseC encoded from the *rpoE-rseABC* operon, and ApbE, each located on three separate loci. [9, 14]. The *rsx* operon is a homolog of *rnfABCDEG* operon that constitutes Rnf in bacteria [1, 4, 9, 16].

RseC and ApbE are partial homologs of the N- and C-terminus of RnfF in *Rhodobacter capsulatus*, respectively, and affect NADH oxidation reaction. [14, 17, 18]. ApbE is a periplasmic FAD-binding lipoprotein and is an enzyme that transfers flavin to membrane proteins [19, 20]. ApbE-mediated vinylation of the Rsx system enables electron transfer required for SoxR reduction [14]. However, it has been suggested that RseC, regulated independently of Rnf in *Clostridium ljungdahlii*, may function as a transcriptional regulator [16]. In *E. coli*, the protein interactions among Rsx proteins and RseC have been identified, indicating potential functional regulation [14]. Therefore, further studies were conducted to elucidate its precise function.

## Materials and Methods

### Bacterial Strains and Plasmids

For the bacterial two-hybrid (BACTH) analysis, we followed the protocol described by

Lee *et al*. [14]. Gene segments were amplified by PCR using primers with appropriate restriction sites and the pKT25-RseC construct as a template. The amplified PCR products were digested with PstI and XbaI, then cloned into pUT18 and pKT25 vectors. Each pair of pUT18-based and pKT25-based constructs was subsequently transformed into the *E. coli* DHP1 strain.

To determine the membrane topology of RseC protein derivatives, PCR-generated gene fragments were cut and ligated in-frame with either the *phoA*’ gene (lacking a signal sequence) or the *lacZ* gene, and subsequently cloned into the pTAC3N plasmid via the multi-cloning site. NdeI and BamHI were used to cut PCR fragments of *rseCN70*, *rseCN98*, and *rseCN130*, while BamHI and NotI were used for PCR fragments of *phoA*’ and *lacZ*.

To overproduce the RseC N-terminal domain, PCR-generated *rseC* gene fragments containing codons for up to 70 amino acids were cloned into pET3a or pET15b (Novagen).

Site-specific mutagenesis of RseC was done with QuikChange Site-Directed Mutagenesis Kit (Stratagene).

### Reporter Enzyme Assays

For LacZ activity assays, GC4468 (F- Δlac U169 rpsL) or MS1343 (GC4468-based *soxS*-*lacZ*) cells harboring recombinant plasmids were grown at 37°C in LB medium containing 50 μg/ml apramycin until reaching an OD_600_ of 0.5. For *phoA* activity assays, CC118 (λpir) cells harboring recombinant plasmids were grown under the same conditions. The β-galactosidase activity was measured by standard method using o-nitrophenyl-β-D-galactopyranoside (ONPG) after permeabilization of cells with SDS-chloroform. For BACTH analysis, DHP1 cells harboring recombinant plasmids were grown at 30°C in LB broth containing 100 μg/ml ampicillin and 50 μg/ml kanamycin with vigorous aeration to OD_600_ of 0.6 before measuring LacZ activity. Alkaline phosphatase activity was measured as described previously [21].

### Purification and Reconstitution of N-Terminal Domain of RseC

*E. coli* BL21-Gold (DE3) cells containing pET-based recombinant plasmids were overexpressed with isopropyl-β-D-thiogalactoside (IPTG) and stored frozen at -80°C in TN100 buffer (50 mM Tris-HCl, pH7.6, 100 mM NaCl). Cell lysates were obtained by sonication from cells and centrifuged for 30 min in microcentrifuge at 4°C. The pellet was washed twice in 20 volumes of TNX0.5 buffer (50 mM Tris-HCl, pH7.6, 100 mM NaCl, 0.5% TritonX-100). The Inclusion body was solubilized with TPU8 buffer (50 mM Tris-HCl, pH7.6, 150 mM KPO4, 8M Urea) to a protein concentration of 2.5 mg/ml, followed by centrifugation at 100,000´g for 1.5 h. The solubilized N70 and N70_C20S_ proteins were renatured by dialyzing against TGDN100 (TN100, 10% glycerol, 1 mM DTT). The refolded proteins were purified through the Q-Sepharose column (Amersham Biosciences, USA). Reconstitution of N70 and N70_C20S_ in the presence of Fe and S was performed as previously described, with some modifications [22] in the anaerobic chamber (Coy laboratory products inc.). GN100 buffer and the deionized water (DW) were purged with N2 gas and kept anaerobic for more than 24 h before use. DTT, FeCl_3_ and Na_2_S powders were dissolved in N2-purged DW to make 10 X stock solutions. Purified N70 protein was equilibrated in an anaerobic atmosphere at 4°C for 24 h in the chamber. It was then incubated with 6- to 8-fold molar excess of Na_2_S and FeCl_3_ in 2.5 ml TGN100 for 4 h at 25°C, followed by elution through PD-10 desalting column with TGDN100 (TGN100, 1-10 mM DTT) to remove small molecules. The absorption spectrum of anaerobically reconstituted RseC was measured at 25°C by scanning 250 – 800 nm range with a UV-VIS spectrophotometer (Shimadzu, Japan) at room temperature in 10 mm path-length Stoppered cuvettes (Agilent Technologies, USA).

### Sensitivity Test

GC4468 and derivative strains were grown for 15 h at 37°C in LB medium containing with or without 50 μg/ml kanamycin for seed culture. After inoculating the seed culture into fresh medium, when the optical density (OD_600_) reaches 0.1 to 0.2, transfer 1% of the culture to prewarmed medium and incubate the remaining culture at 37°C for an additional 6 h. After 6 h at 37°C, the OD_600_ of the 1% inoculated samples will reach 0.5 to 0.8, while the continuously grown samples will reach an OD_600_ of 3.0 or higher. The former is considered the exponential phase sample, and the latter is the stationary phase sample. Each sample is diluted to an OD_600_ of 0.5 with LB medium, and four serial 5-fold dilutions are prepared. These dilutions are then spotted onto LB solid media containing 0.32 mM cumene hydrogen peroxide (CHP) or 0.42 mM hydrogen peroxide (H_2_O_2_).

### Catalase Activity Assay of RseC

The exponential phase and stationary phase samples of GC4468 and Δ*rseC* were harvested by centrifugation for 10 min at 4°C. The harvested cells were resuspended in 50 mM potassium phosphate buffer. Cell lysates were obtained by sonication, and cell debris was removed by centrifugation for 30 min at 4°C. The protein concentration was determined using the Bio-Rad protein assay. Twenty micrograms of proteins in cell extracts were loaded onto a 7% native PAGE. The gel was incubated with 4 mM H_2_O_2_ for 10 min, briefly rinsed twice, and then a dark green background color developed in a solution of 2% potassium ferricyanide and 2% ferric chloride under illumination.

## Results

### Configuration and Membrane Topology of RseC

**Fig. 1:**
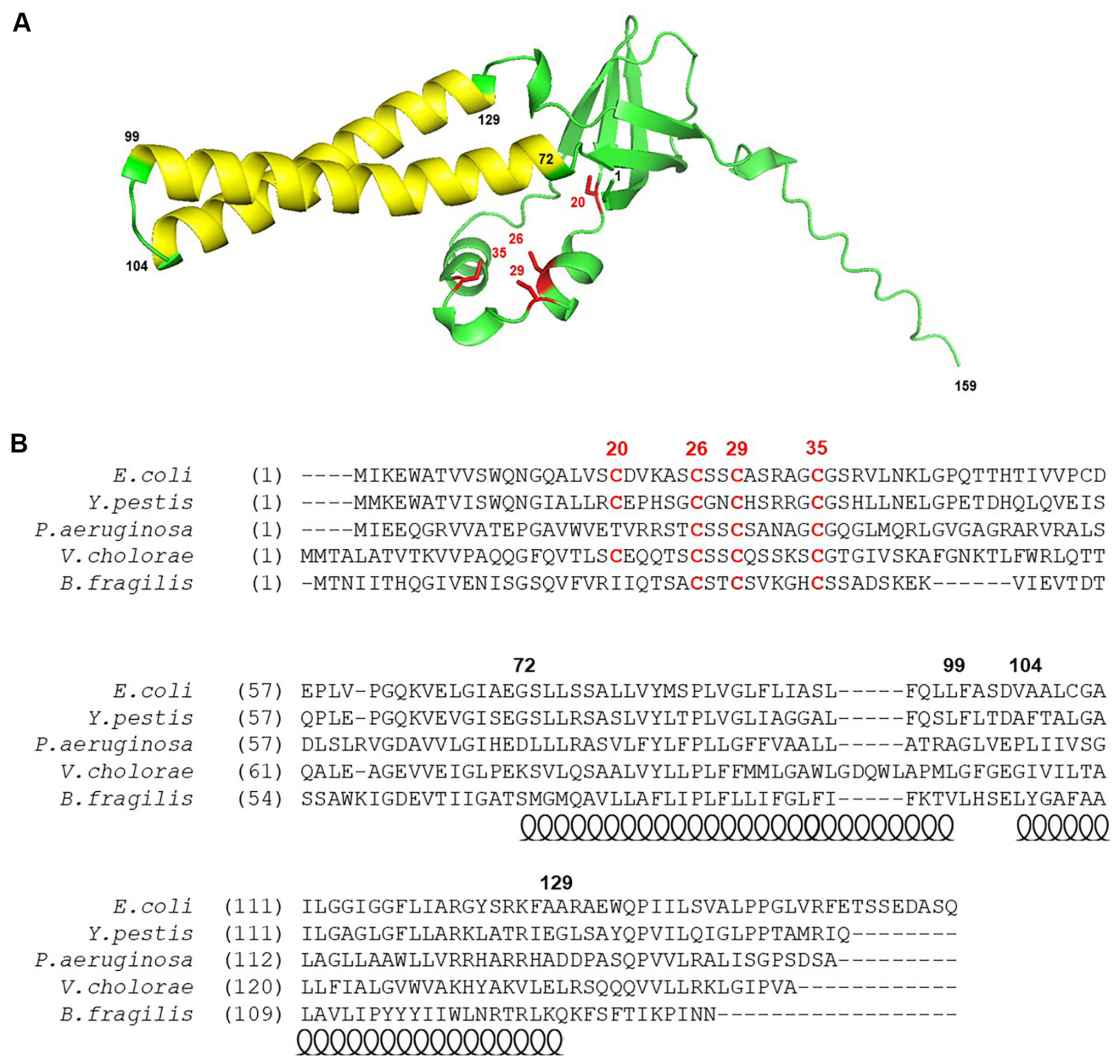
RseC Homologs. (**A**) Structure of RseC in *E. coli* predicted by AlphaFold. Red indicates conserved cysteines. Numbers represent the start or end positions of predicted secondary structures or cysteines. (**B**) Amino acid sequence comparison of RseC in *E. coli*, *Y. pestis*, *P. aeruginosa*, *V. cholerae*, and *B. fragilis*. Circles indicate hydrophobic alpha helices.

Genetic analysis using the bacterial two-hybrid system determined that both the N-terminal and C-terminal domains of RseC are located in the cytoplasm [14]. Predictions using TMHMM and AlphaFold [23, 24] corroborated these findings (Fig. 1A). The characteristic alpha-helical region forms the first transmembrane helix from amino acids 72 to 99 of the RseC protein, and the second transmembrane helix from amino acids 104 to 129. The N-terminal cytoplasmic domain and the C-terminal cytoplasmic domain are in close proximity and interact with each other. The N-terminal cytoplasmic domain contains four cysteine residues, three of which are well conserved. Among the homologs of *E. coli* RseC with high sequence similarity, the first cysteine is not conserved in *Pseudomonas aeruginosa* and *Bacteroides fragilis*, but it is conserved in *Yersinia pestis* and *Vibrio cholerae* (Fig. 1B). The conserved residues at positions 26, 29, and 35 are clustered together, while the first residue at position 20 is relatively distant.

**Fig. 2:**
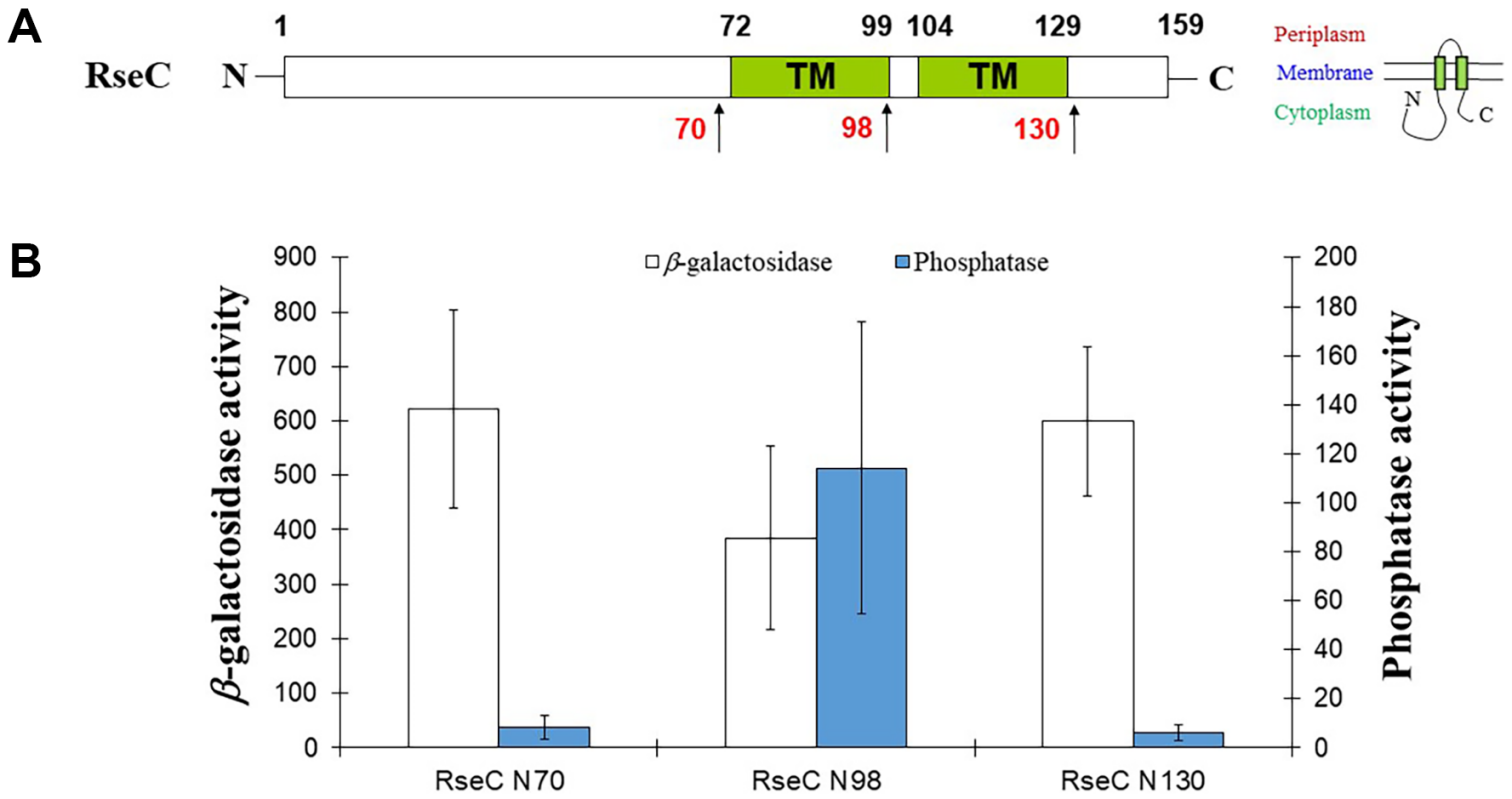
Topology of RseC. (**A**) Schematic representation of RseC. Numbers indicate the start or end positions of predicted secondary structures. TM in green boxes indicate transmembrane helices, and red numbers represent translational fusion sites with LacZ or PhoA. (**B**) Activity assay performed with translationally fused truncated RseC (RseC N70, RseC N98, RseC N130) with LacZ or PhoA. β-galactosidase activity is represented by empty bars (left y-axis), and phosphatase activity is represented by filled bars (right y-axis). The graph is based on the average of at least three independent experiments, with error bars indicating standard deviation.

Even in AlphaFold, the accuracy is low for the cysteine-rich region and the ends of the transmembrane helix. Additionally, the transmembrane helix is represented differently depending on the prediction tool, necessitating further verification. We performed translational fusion of β-galactosidase (LacZ) and alkaline phosphatase (PhoA) to RseC, either at the C-termini or at specific positions near the membrane-targeting motifs. N70 was selected as a representative for the region predicted to be solely in the cytoplasmic domain, N98 for the region consisting of only the first transmembrane helix, and N130 for the region fully containing two transmembrane helices (Fig. 2A).

The strong phosphatase activity in N98 and the high β-galactosidase activity in N70 and N130 suggest that both the N-terminal and C-terminal domains are located in the cytoplasm. Reporter enzyme activity measurements indicate that RseC likely positions its N- and C-terminal domains flanking the two TM motifs in the cytoplasm (Fig. 2B)

### Complementation and Interaction of RseC Fragments

**Fig. 3:**
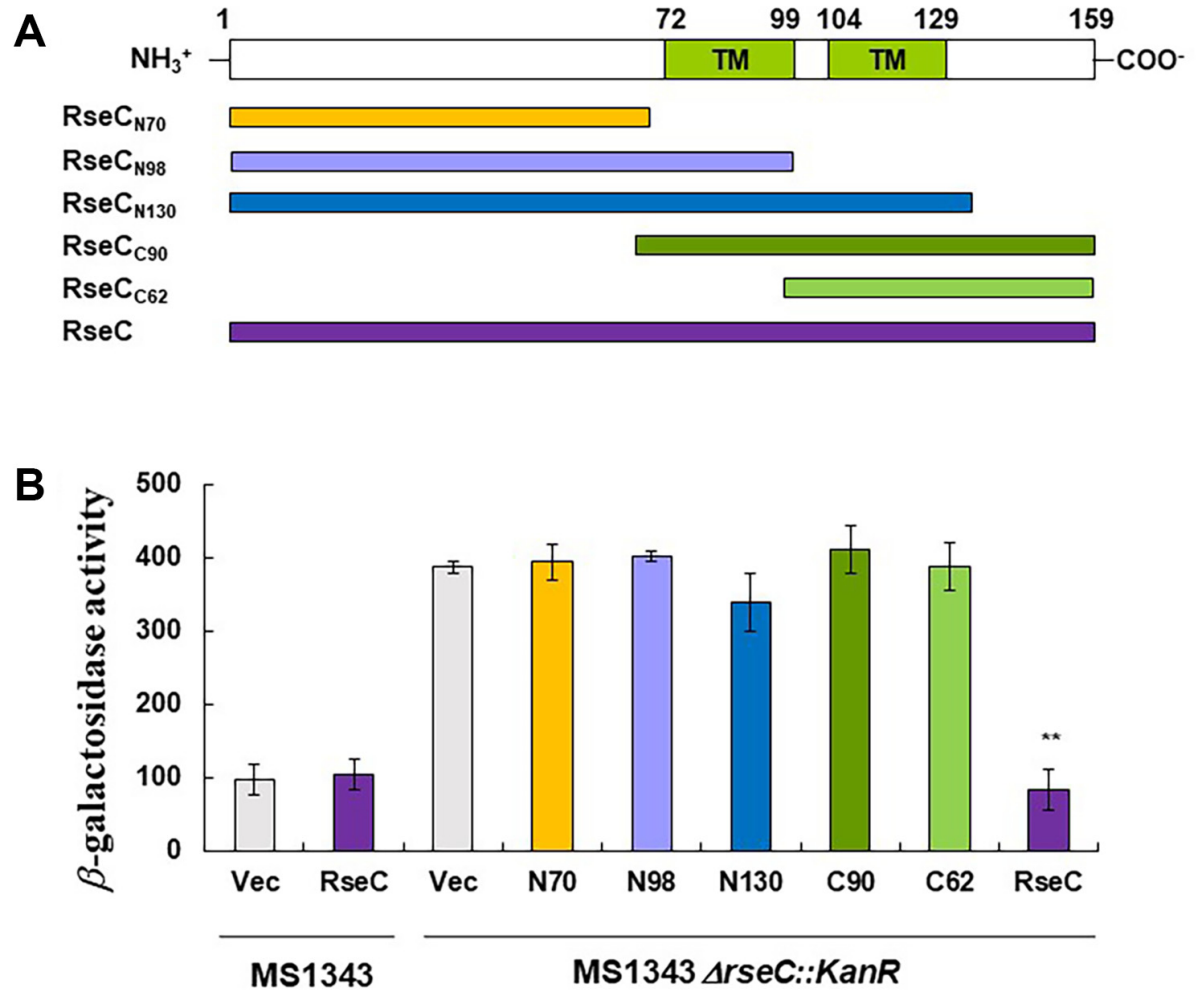
Complementation of RseC fragments. (**A**) Schematic representation of RseC. Numbers indicate the start or end positions of predicted secondary structures. TM in green boxes indicate transmembrane helices. The names of the truncated forms of RseC are indicated by the end position of the amino acid sequence and the number of amino acids. The full-length RseC sequence is shown in purple, N70 in yellow, N98 in violet, N130 in blue, C90 in green, and C62 in light green. (**B**) Each RseC fragment in the pTAC3N vector was complemented in MS1343 and its *rseC* mutant strain. Each bar represents the average β-galactosidase activity from at least three independent experiments, with error bars indicating standard deviation. ** indicates *p* < 0.005 compared to vector complementation.

To elucidate the functional significance of RseC structure, we examined whether sequences present in the cytoplasm and inner membrane could complement SoxR reducer function. We expressed the full-length RseC, the N-terminal cytoplasmic domain (N70), the first membrane helix (N98), both transmembrane domains (N130), and the C-terminal truncations (C90 and C62) using the expression vector pTAC3N (Fig. 3A). The functional complementation of each segment was assessed by measuring the expression levels of P*soxS*-*lacZ* in MS1343, which is activated by SoxR oxidation. Overexpression of RseC in its native context did not result in growth defects or impair the function of the SoxR reducer complex, as confirmed by the beta-galactosidase assay. To further verify functional complementation, we measured the expression levels of P*soxS*-*lacZ* in the *rseC* mutant strain. Only the full-length RseC was able to restore the reduced SoxR activity (Fig. 3B).

Previous genetic analysis using the bacterial two-hybrid system predicted that RseC interacts with electron transfer structures such as RsxB and RsxD of the SoxR reducer complex at the cytoplasmic membrane. The bacterial two-hybrid system measures interaction by forming a single domain from two split β-galactosidase fragments located in the cytoplasm. To identify the regions involved in these interactions, we performed translational fusions of partial LacZ domains to the C-terminal parts of N70, N98, N130, C90, and C62, and the remaining partial LacZ domains to RseC, RsxB, and RsxD (Table 1). The basal activities of LacZ in the DHP1 strain transformed with two parental vectors ranged from 71 to 138 Miller units. N130, which contains the cysteine-rich region and two transmembrane helices, showed more than double the β-galactosidase activity, similar to the full-length RseC protein. This indicates that the C-terminal cytoplasmic domain is not required for interaction with the SoxR reducer complex. The lack of interaction observed in C90, which includes two transmembrane helices and the C-terminal part but lacks the cysteine-rich region, suggests that the N-terminal cytoplasmic domain also plays a crucial role in the interaction.

**Fig. 4:**
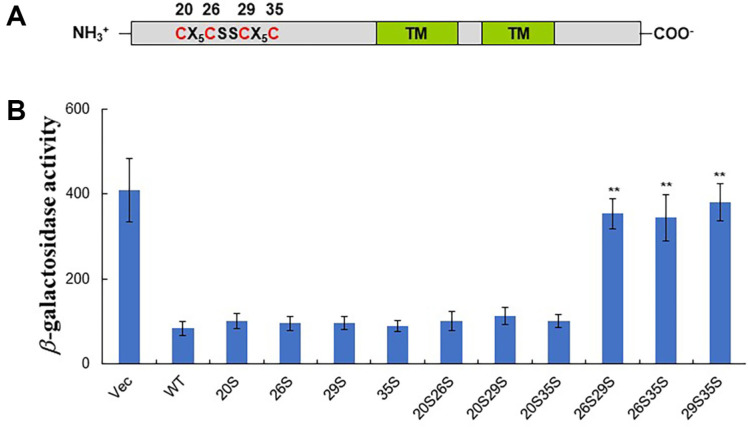
Complementation of RseC cysteine substituents. (**A**) Schematic representation of RseC. Numbers indicate the positions of cysteines. TM in green boxes indicate transmembrane helices. (**B**) Each RseC cysteine-to-serine substitution construct in the pTAC3N vector was complemented in the MS1343 *rseC* mutant strain. Each bar represents the average β- galactosidase activity from at least three independent experiments, with error bars indicating standard deviation. ** indicates *p* < 0.005 compared to wild-type complementation.

The N-terminal cytoplasmic region of RseC contains three well-conserved cysteines at positions 26, 29, and 35, and a less conserved cysteine at position 20 (Fig. 4A). Structural predictions indicated that the three conserved cysteines are likely to be in close proximity. Given the potential functional importance of these closely located cysteines, we measured SoxR reducer activity using full-length RseC proteins in which each cysteine was individually substituted with serine. The cysteine at position 20 was found to be unrelated to SoxR reducer function, while the SoxR reducer activity was lost only when two of the three conserved cysteines were substituted (Fig. 4B). These results indicate that RseC requires at least two cysteines to perform its function.

### The Cytoplasmic Domain of RseC and Oxygen Sensitive Fe-S Cluster

**Fig. 5:**
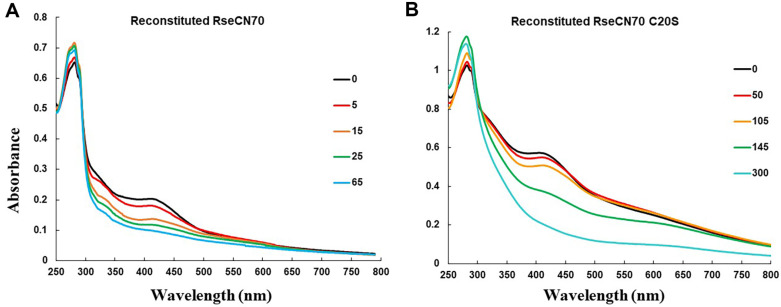
UV-VIS Absorption Spectra of the Cytoplasmic Domain of RseC. (**A**) the reconstituted N70 protein was monitored from 250 nm to 800 nm immediately after reconstitution (t = 0, black) and after various durations of air exposure (t = 5 (red), 15 (orange), 25 (green), and 65 (cyan) min) at room temperature. (**B**) the reconstituted N70 protein with the 20^th^ cysteine substituted by serine was monitored from 250 nm to 800 nm immediately after reconstitution (t = 0, black) and after various durations of air exposure (t = 50 (red), 105 (orange), 145 (green), and 300 (cyan) min) at room temperature.

To investigate the role of cysteines, we obtained the N70 protein of RseC and conducted experiments related to cysteine involvement. Overproduction of N70 in *E. coli* yielded inclusion bodies with reddish brown color, which were solubilized in 8 M urea. We attempted reconstitution of the Fe-S cluster by incubating N70 in reconstitution buffer containing Fe^2+^ and sulfide at room temperature in an anaerobic chamber. The UV-VIS absorption spectrum of the reconstituted protein exhibited a single broad peak at 420 nm, consistent with an absorption profile of [4Fe-4S] cluster. When 9.3 μM of anaerobically reconstituted N70 in 0.5 mM DTT was exposed to air, the absorption spectra changed over time, suggesting a relatively rapid degradation of the [4Fe-4S] cluster through oxidation (Fig. 5A). The substitution of unconserved cysteine did not affect the formation of the oxygen-sensitive [4Fe-4S] cluster. 12.3 μM of reconstituted N70_C20S_ proteins in 1 mM DTT showed a slower degradation of the [4Fe-4S] cluster through oxidation (Fig. 5B). Purified proteins that contain Trp, Tyr residues, or Cys-Cys disulfide bonds exhibit absorbance at 280 nm. The N70 protein contains 2 Trp and 5 Cys residues. The increase in A280 absorbance is reported to occur during the oxidation of proteins [25], indicating that N70 is prone to oxidation upon exposure to oxygen. While the rate of oxidation may vary depending on the concentration ratio of DTT to reconstituted RseC derivatives, it was generally similar.

### The Physiological Role of RseC

**Fig. 6:**
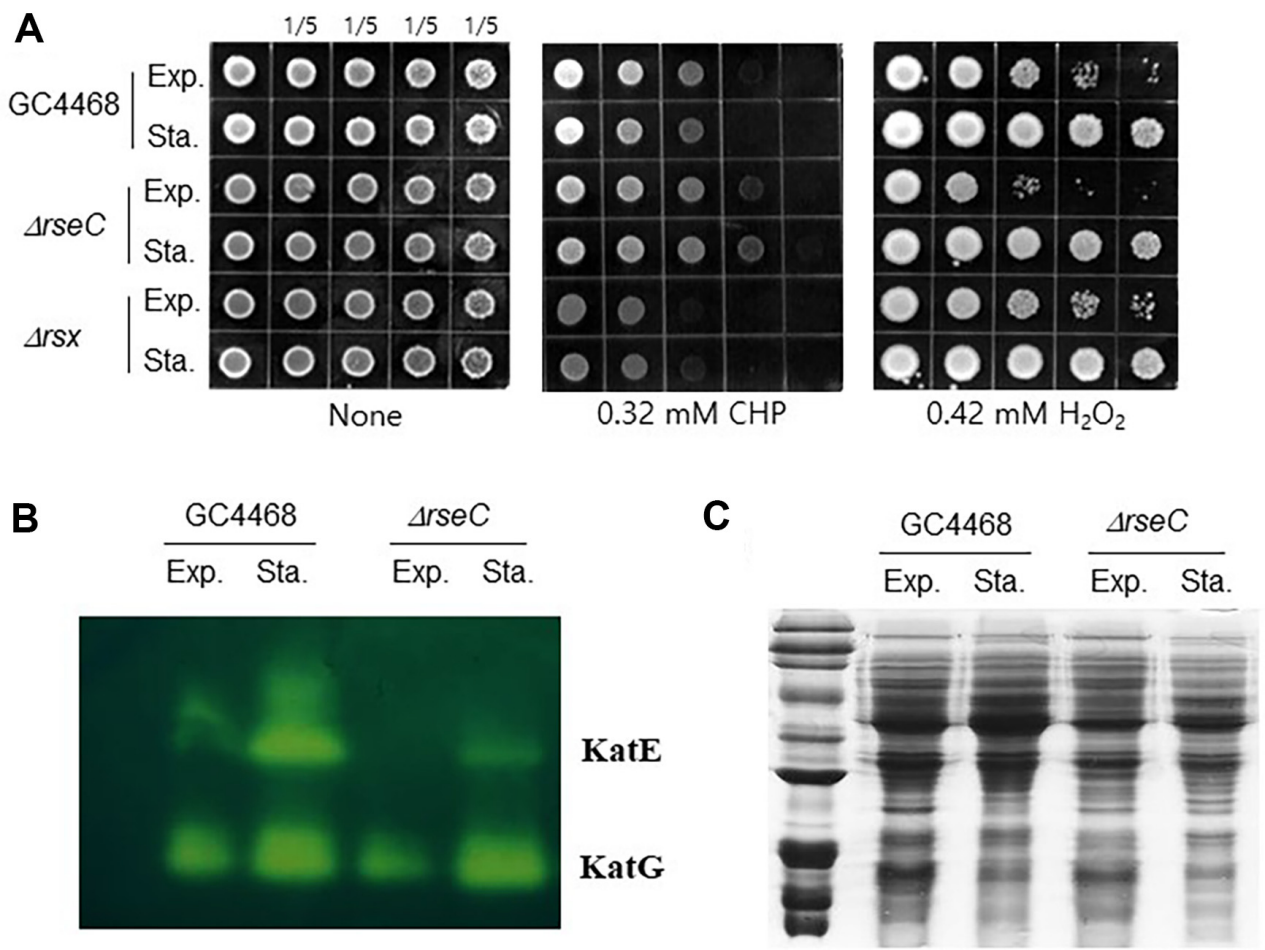
Physiological function of RseC. (**A**) Sensitivity to cumene hydrogen peroxide (CHP) and hydrogen peroxide was measured for GC4468, *rseC* mutant, and *rsx* mutant strains during exponential and stationary phases. The starting OD600 was 0.5, and each spot represents a 5-fold dilution. (**B**) Catalase activity assay for GC4468 and *rseC* mutant strains during exponential and stationary phases. (**C**) Comparison of intracellular protein expression patterns in GC4468 and *rseC* mutant strains during exponential and stationary phases.

RseC, which is subject to different transcriptional regulation in an orphan locus and exists as a membrane protein with oxygen-sensitive conformational changes in its cytoplasmic domain, suggests that it may function independently and possess roles other than being a subunit of the SoxR reducer. To verify independent redox-related functions, we examined its role in reactive oxygen species. Since the SoxRS regulon is directly involved in defense mechanisms against redox cycling chemicals or superoxide, it is difficult to compare these activities with those of the SoxR reducer. Instead, we tested the reactivity to the organic peroxide CHP and the membrane-permeable reactive oxygen species H_2_O_2_. To confirm the lack of direct relationship with SoxRS, we compared samples from the stationary phase and exponential phase. Resistance to CHP was observed to be approximately 5-fold higher in the stationary phase, while sensitivity to hydrogen peroxide was about 5-fold higher in the exponential phase (Fig. 6A). To investigate the cause of these changes in cellular homeostasis, we measured the activity of catalases, which could be directly responsible. We found that catalase activity was slightly reduced in the *rseC* mutant, with KatG activity decreased in the exponential phase and KatE activity slightly reduced in the stationary phase (Fig. 6B). When analyzing the overall protein profiles using SDS-PAGE, we could not exclude the influence of the SoxRS regulon (Fig. 6C).

## Discussion

Research has focused on the role of RseC in maintaining homeostasis following strong oxidative reactions induced by redox cycling agents and superoxide anion radicals through the SoxRS system in *E. coli* [9, 11, 12, 14, 26-32]. The absence of RseC in actinomycetes, which possess SoxR, and its presence in anaerobic bacteria lacking SoxR, suggests that the function of RseC as a SoxR reducer is not well conserved [11, 28, 31, 33]. Studies have shown that Rnf can be assembled without RseC in various bacteria, such as *V. cholerae* and *Acetobacterium woodii* [34, 35]. Additionally, genetic analysis in *C. ljungdahlii* has revealed that RseC is involved in the transcriptional regulation of the rnf operon [16]. The multifunctionality of RseC is likely due to its poorly conserved nature.

In *R. capsulatus*, the homolog of RseC is identified as RnfF. RnfF had two cysteine motives, CX_2_CX_5_C, CX_3_CXCX_2_C, which worked ligands in cytoplasm. The former was conserved in RseC. The latter was not conserved in any ApbE homologs. RseC contains a conserved cysteine motif, CX_5_CX_2_CX_5_C, in the N-terminal domain in *E. coli* [9]. The first cysteine is not conserved and is not utilized in the function of the SoxR reducer complex, suggesting that the conserved motif should be considered as CX_2_CX_5_C. These closely conserved cysteine motifs are often found in structures forming disulfide bonds or Fe-S clusters in ferredoxin or Fe-S cluster containing enzymes. Experiments using only the cytoplasmic domain have confirmed that it can form an oxygen-sensitive Fe-S cluster. Typically, 3 or 4 cysteines are required to form a stable Fe-S cluster [36]. In *E. coli*, it has been confirmed that 2 out of the 3 conserved cysteines are essential for function, indicating that all three cysteines are involved in Fe-S cluster formation, but a stable structure is achieved with the help of other factors.

When the RseC protein is expressed with only the N-terminal cytoplasmic domain, it is mostly observed in inclusion bodies. The structural instability and oxygen sensitivity of the conserved cysteine motif in RseC appear contradictory to its function related to reactive oxygen species. However, the structure predicted by AlphaFold, where the C-terminal cytoplasmic region interacts with the N-terminal domain, suggests that the instability of the cysteine motif observed when only the N-terminal cytoplasmic domain is expressed might differ in actual cellular conditions. The results indicating that only the full-length sequence can functionally complement support this hypothesis.

In *E. coli*, RseC is co-expressed with the sigma E operon, a master regulator of cell envelope stress, but its membrane-related functions have not been identified so far. Similarly, in *P. aeruginosa*, the function of MucC within the *algU*-*mucA*-*mucB*-*mucC*-*mucD* operon, which regulates alginate production, remains unclear. SoxR reducer mutants sequentially regulate the MarA/SoxS regulon, which shares the mar box, by activating SoxS through SoxR [37]. This simultaneous activation of antibiotic resistance, oxidative stress defense regulon, and homeostasis maintenance genes makes it difficult to pinpoint an independent additional function of RseC. The physiological characteristics observed in other *rseC* mutant, such as increased intracellular iron and decreased NADPH, are intertwined with the activation caused by SoxR oxidation and the resultant effects of the SoxRS regulon [38]. This physiological trait supports the conclusion that these mutants are sensitive to H_2_O_2_, thereby corroborating the findings of this study [38]. This study has demonstrated that the conserved cysteine motifs in both RseC and MucC are involved in the formation of Fe-S clusters and influence factors present in the cell membrane, thereby altering the response to reactive oxygen species passing through the membrane. These changes can affect the physiological characteristics of the cell. Additionally, Fe-S clusters in membrane proteins are closely associated with electron transport systems, suggesting that future research could observe membrane changes induced by RseC proteins, laying the groundwork for further studies.

## Figures and Tables

**Table 1:** Bacterial two-hybrid analysis of interactions between pairs of SoxR reducer components and RseC fragments^a^.

pUT18	N70	N98	N130	C90	C62	RseC
pKT25						
RseC	150 ± 55	145 ± 31	**351 ± 57**	143 ± 50	130 ± 32	**270 ± 97**
RsxB	122 ± 21	111 ± 32	**271 ± 91**	128 ± 28	106 ± 19	**234 ± 39**
RsxD	113 ± 26	124 ± 42	**762 ± 88**	134 ± 36	93 ± 12	**347 ± 48**

^a^Average values were presented with standard deviations obtained from three independent experiments. Values that exceed 2-fold increase (*p* < 0.05) were highlighted in bold.
